# The role of *TFPI2* hypermethylation in the detection of gastric and colorectal cancer

**DOI:** 10.18632/oncotarget.21097

**Published:** 2017-09-20

**Authors:** Haochang Hu, Xiaoying Chen, Cheng Wang, Yuting Jiang, Jingjing Li, Xiuru Ying, Yong Yang, Bin Li, Cong Zhou, Jie Zhong, Dongping Wu, Jieer Ying, Shiwei Duan

**Affiliations:** ^1^ Medical Genetics Center, School of Medicine, Ningbo University, Ningbo, Zhejiang 315211, China; ^2^ Department of Medical Oncology, Shaoxing People's Hospital, Shaoxing Hospital of Zhejiang University, Zhejiang 312000, China; ^3^ Department of Medical Oncology, Zhejiang Cancer Hospital, Hangzhou, Zhejiang 310022, China

**Keywords:** tissue factor pathway inhibitor 2, DNA methylation, gastric cancer, colorectal cancer, quantitative methylation-specific polymerase chain reaction

## Abstract

Gastrointestinal cancer is a prevalent disease with high morbidity and mortality. Tissue factor pathway inhibitor 2 (*TFPI2*) gene could protect the extracellular matrix of cancer cells from degradation and tumor invasion. The goal of our study was to estimate the diagnostic value of *TFPI2* hypermethylation in gastric cancer (GC) and colorectal cancer (CRC). *TFPI2* methylation was measured by quantitative methylation-specific polymerase chain reaction (qMSP) method in 114 GC and 80 CRC tissues and their paired non-tumor tissues. Our results showed that *TFPI2* methylation was significantly higher in tumor tissues (GC: 29.940% vs. 12.785%, *P* < 0.001; CRC: 26.930% vs. 5.420%, *P* < 0.001). The methylation level of *TFPI2* in colorectal tumor tissues was significantly higher than that in colorectal normal tissues (26.930% versus 0.002%, *P* < 0.00001). In GC, *TFPI2* hypermethylation yielded an area under the curve (AUC) of 0.762 (95% CI: 0.696–0.828) with a sensitivity of 68% and a specificity of 83%. In CRC, *TFPI2* hypermethylation yielded an AUC of 0.759 (95% CI: 0.685–0.834) with a sensitivity of 61% and a specificity of 84%. Similarly, TCGA data also supported *TFPI2* hypermethylation was a promising diagnostic marker for GC and CRC. Moreover, the dual-luciferase reporter assay showed *TFPI2* fragment could upregulate gene expression (fold change = 5, *P* = 0.005). Data mining further indicated that *TFPI2* expression in CRC cell lines was significantly increased after 5’-AZA-deoxycytidine treatment (fold change > 1.37). In conclusion, *TFPI2* hypermethylation might be a promising diagnostic biomarker for GC and CRC.

## INTRODUCTION

The gastrointestinal tract is one of the most predilection sites of tumorigenesis [1]. In China, about 679,100 cases of gastric cancer (GC) and 376,300 cases of colorectal cancer (CRC) were diagnosed in 2015 [2]. Despite the rapid improvement in clinical therapy, GC and CRC are still global public health problems due to their poor prognosis [3, 4]. Lacking a gold standard of noninvasive method is considered as the major obstacle to the screening, diagnosis and individualized treatment of GC and CRC. For this purpose, it is urgent to identify suitable biomarkers for the early detection of GC and CRC.

The development of GC and CRC is characterized by the dysregulation of genetic, epigenetic, and environmental factors [5, 6]. As a bridge between the genetic and environmental factors, epigenetic modification plays an important role in cancer initiation and progression. DNA methylation is acknowledged as the principle mechanism of dysfunction of tumor suppressor genes [7] and the activation of oncogenes [8]. DNA methylation has been widely studied in GC and CRC [9–12].

Tissue factor pathway inhibitor 2 (*TFPI2*) gene is located on chromosome 7q22, encoding a Kunitz-type serine proteinase inhibitor [13]. *TFPI2* could protect the extracellular matrix of cancer cells from degradation and tumor invasion [14]. The loss of *TFPI2* function might enhance the invasive potential of neoplastic cells in several cancers [15]. *TFPI2* hypermethylation has been observed in CRC [16–18], GC [19–21], hepatocellular carcinoma [22], pancreatic cancer [23], and cervical cancer [15]. Interestingly, *TFPI2* methylation disappeared in the serum of CRC patients after curative surgery [24], indicating a close correlation between *TFPI2*methylation and CRC occurrence.

In this study, we used a quantitative methylation specific PCR (qMSP) method to measure *TFPI2* methylation in GC and CRC tissues of Chinese Han patients, since the low sensitivity and specificity in the MSP method was still the main obstacle to the clinical application of *TFPI2* methylation [16, 25]. The aim of this study was to evaluate whether cytosine-phosphate-guanine (CpG) island methylation of *TFPI2* could serve as a valuable biomarker in GC and CRC.

## RESULTS

**Figure 1:**
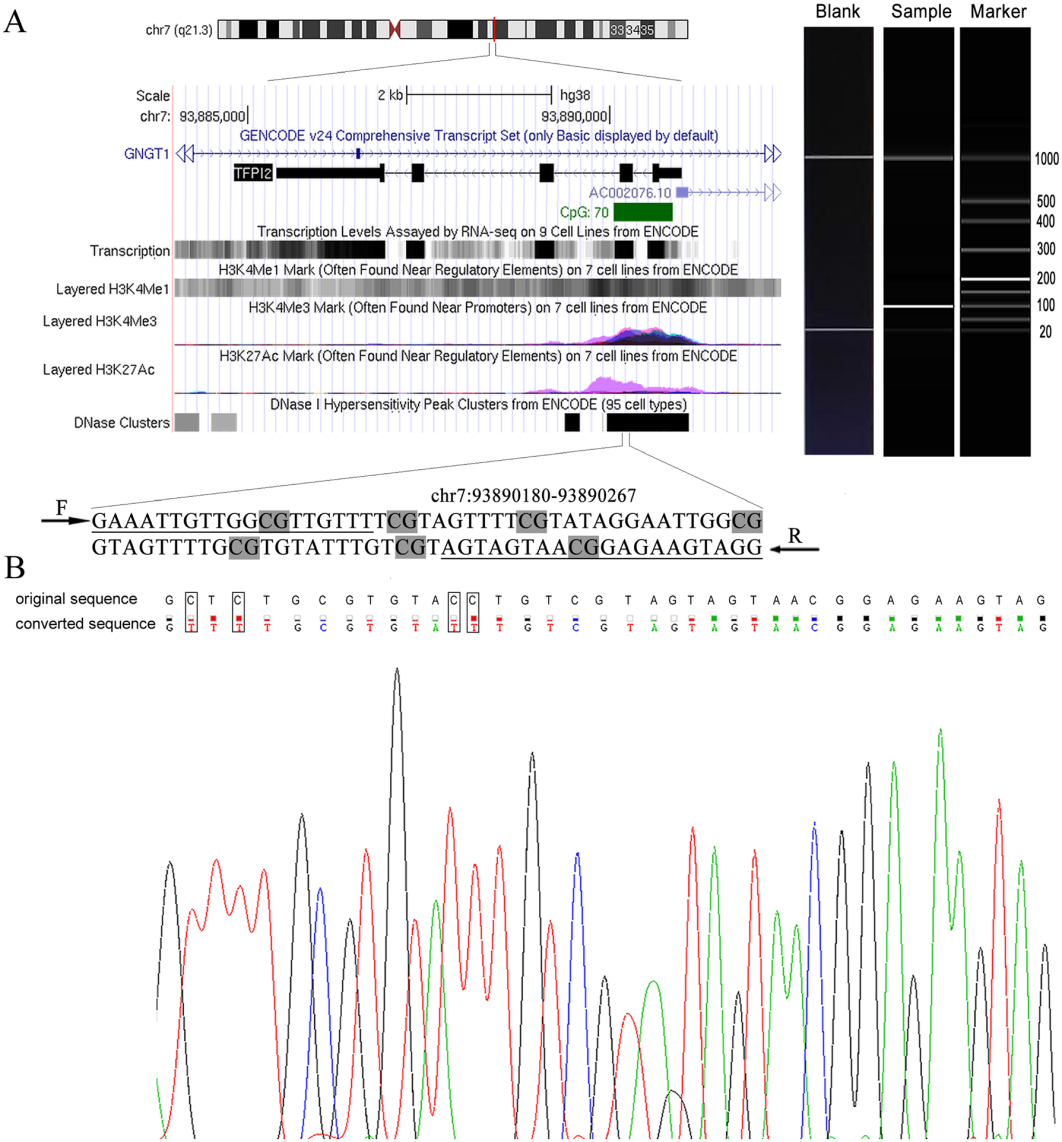
Target sequences on *TFPI2* CpG island (CGI) region (**A**) The genomic position and functional annotation of amplified fragment from UCSC genome browser according to human 2013 (GRCh38/hg38) assembly. The qMSP primers were underlined and seven CpG sites were in grey. F: forward primer; R: reverse primer. The picture on the right was the electrophoresis result of a representative qMSP product. (**B**) The top row of the sequence represented the original sequence, and the second row showed the converted sequence. And the framed base indicated that the cytosines were replaced by thymines (C to T conversion) in bisulfite-treated DNA.

In the current study, we recruited 114 GC patients, 80 CRC patients and 22 non-tumor individuals to investigate the role of *TFPI2* methylation on the detection of GC and CRC. There were two Methyl450 CpG sites (cg12973591 and cg22799321) located in the tested fragment (88 bp, hg38, chr7:93890180-93890267, Figure 1A). Meanwhile, Sanger sequencing showed that the amplified fragment matched the target sequence (Figure 1B).

**Figure 2:**
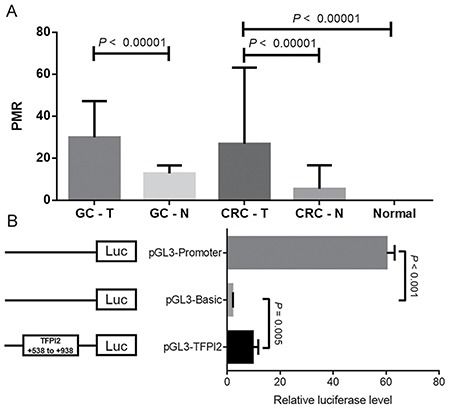
(**A**) Comparisons of *TFPI2* methylation levels between tumor tissues and paired adjacent non-tumor tissues in GC patients and CRC patients. (**B**) Dual-luciferase reporter assay in HEK-293T cell line. The pGL3-Basic and pGL3-Promoter vectors were used as negative and positive controls, respectively. The pGL3-TFPI2 stood for the recombinant *TFPI2* fragment ligated to pGL3-Basic vector. Relative luciferase activity was performed in quadruplicate. T stands for tumor tissues; N stands for adjacent non-tumor tissues. Normal stands for colorectal normal tissues from normal persons. Statistical values and the bar were presented as median with interquartile range.

Our data showed that *TFPI2* methylation in tumor tissues was significantly higher than that in paired adjacent tissues [GC: 29.940% (15.472%, 47.295%) versus 12.785% (9.678%, 16.575%), P < 0.001; CRC: 26.930% (8.478%, 63.145%) versus 5.420% (1.345%, 16.638%), P < 0.00001; Figure 2A]. The methylation levels of *TFPI2* in colorectal tumor tissues were significantly higher than those in colorectal normal tissues [26.930% (8.478%, 63.145%) versus 0.002% (0.001%, 0.054%), *P* < 0.00001, Figure 2A]. These data supported the previous findings that *TFPI2* hypermethylation could be a potential novel biomarker for GC and CRC [18]. Then, *TFPI2* hypermethylation was found in 85 out of 114 GC tissues and 61 out of 80 CRC tissues. *TFPI2* hypermethylation was a risk factor for GC and CRC [GC: OR = 8.591 (4.733–15.593); CRC: OR = 10.307 (4.976–21.351)]. Subsequently, we examined the correlation between *TFPI2* methylation and the clinicopathological features of GC and CRC patients. However, no statistically significant correlation was found with age, gender, differentiation, lymph node metastasis, stage and tumor size (Table 1). We also compared the *TFPI2* methylation levels between GC and CRC tumor samples, and no significant difference was found (*P* = 0.569). Our results didn't support *TFPI2* hypermethylation as a differential biomarker between GC and CRC.

**Table 1:** Association of *TFPI2* methylation with clinical characteristics in gastric cancer and colorectal cancer patients

Clinical characteristics	Variable	Gastric cancer patients		Colorectal cancer patients	
		Numbers, PMR	*P* Value	Numbers, PMR	*P* Value
Age	≤ 60	66, 29.485 (16.668,47.478)	0.754	42, 29.845 (8.565,49.010)	0.343
	> 60	48, 31.685 (14.290,48.015)		38, 23.720 (8.300,94.095)	
Gender	Male	78, 28.260 (15.125,48.570)	0.804	51, 23.260 (6.990,52.010)	0.18
	Female	36, 32.725 (16.538,46.143)		29, 34.390 (12.680,65.935)	
Differentiation	Poorly differentiated	67, 31.020 (16.270,45.770)	0.653	15, 11.460 (4.130,48.770)	0.209^a^
	Moderately + Well differentiated	47, 29.700 (14.600,53.890)		48, 32.160 (11.280,65.990)	
Disease stage	I + II	18, 20.935 (9.700,38.138)	0.126	41, 24.460 (9.945,53.230)	0.867
	III + IV	96, 32.050 (16.913,48.495)		39, 27.530 (6.990,67.350)	
Tumor Size	≤ 6 cm	69, 29.860 (15.415,52.230)	0.754	58, 23.120 (6.753,53.160)	0.202
	> 6 cm	45, 30.020 (15.435,45.660)		22, 34.930 (13.298,68.030)	
Lymph nodes metastasis	Positive	97, 31.020 (16.535,47.670)	0.599	43, 32.580 (10.910,74.110)	0.193
	Negative	17, 22.980 (11.585,48.595)		37, 22.980 (6.300,48.650)	

PMR stands for the percentage of methylated reference, and data were presented as median (interquartile range). *P* value is calculated by Spearman test. a: The information of two cases’ differentiation was lost.

**Figure 3:**
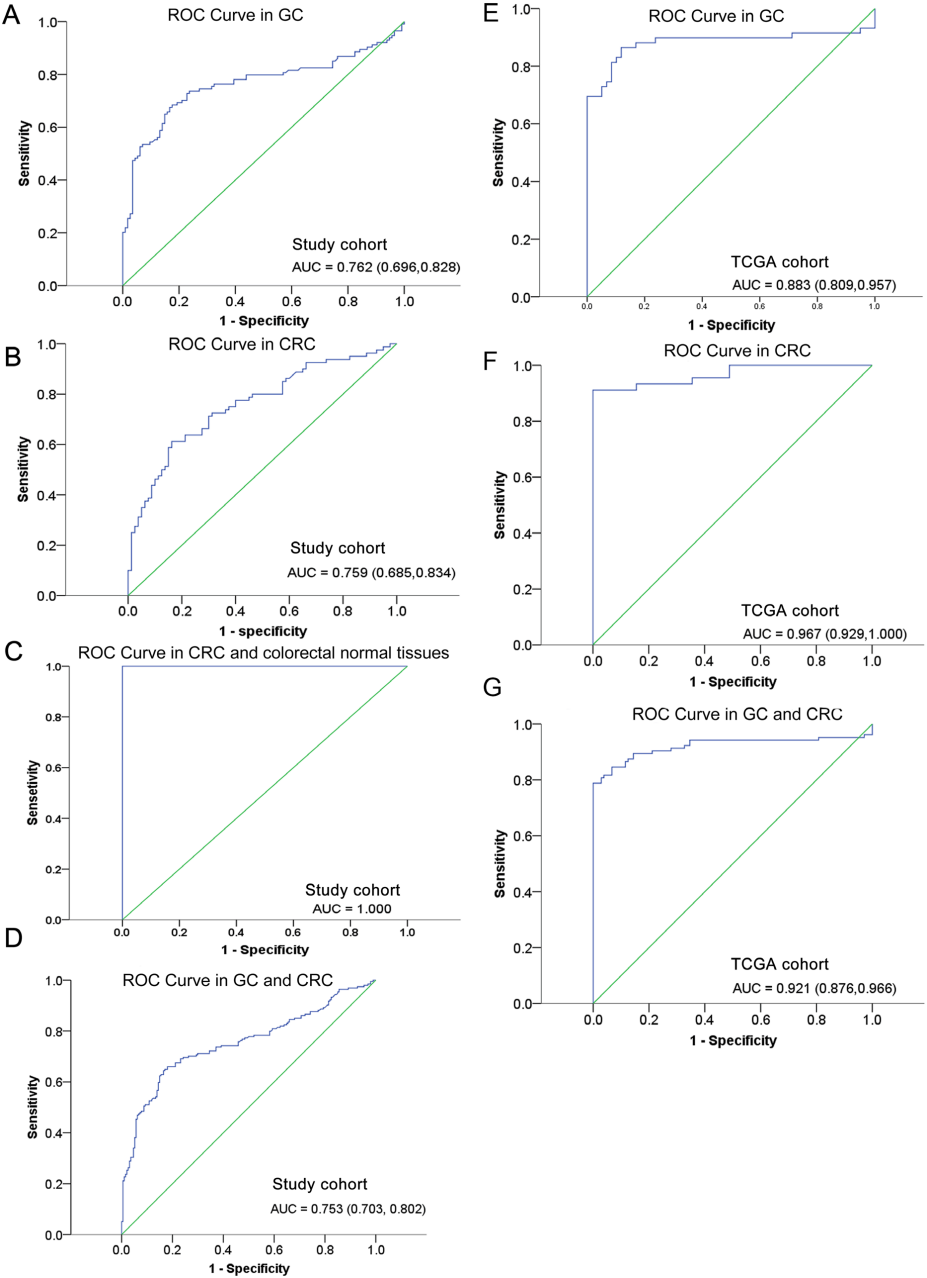
ROC curves for the diagnostic value of *TFPI2* hypermethylation in (**A**) GC patients, (**B**) CRC patients, (**C**) CRC and colorectal normal tissues, and (**D**) combined individuals from study cohort, and (**E**) GC patients, (**F**) CRC patients, (**G**) combined individuals from TCGA cohort. ROC: receiver operating characteristic. AUC: area under the curve.

Since *TFPI2* methylation was different in GC and CRC patients (85/114 in GC and 61 /80 in CRC), we separately estimated the diagnostic value of *TFPI2* methylation in GC and CRC. In GC, *TFPI2* hypermethylation yielded an area under the curve (AUC) of 0.762 (95% CI: 0.696–0.828) with a sensitivity of 68%, a specificity of 83%, a positive predictive value (PPV) of 74%, and a negative predictive value (NPV) of 74% (Figure 3A). In CRC, *TFPI2* hypermethylation yielded an AUC of 0.759 (95% CI: 0.685–0.834) with a sensitivity of 61%, a specificity of 84%, a PPV of 76%, and a NPV of 76% (Figure 3B). Notably, *TFPI2* hypermethylation yielded a high AUC of 1.00 (sensitivity: 100%; specificity: 100%) in CRC by comparing with the methylation level of *TFPI2* between CRC and colorectal normal tissues (Figure 3C). Moreover, *TFPI2* hypermethylation yielded a combined AUC of 0.753 (95% CI: 0.703–0.802) with a sensitivity of 66% and a specificity of 82% (Figure 3D).

Similarly, TCGA data also supported *TFPI2* hypermethylation as a promising diagnostic marker in GC and CRC. In GC, *TFPI2* hypermethylation yielded an AUC of 0.883 (95% CI: 0.809–0.957) with a sensitivity of 86% and a specificity of 89% (Figure 3E). In CRC, *TFPI2* hypermethylation yielded an AUC of 0.967 (95% CI: 0.929–1.000) with a sensitivity of 91% and a specificity of 100% (Figure 3F). Moreover, *TFPI2* hypermethylation yielded a combined AUC of 0.921 (95% CI: 0.876–0.966) with a sensitivity of 79% and a specificity of 100% (Figure 3G).

In order to check whether *TFPI2* CpG island region (+538 bp to +938 bp) was able to regulate gene expression, we performed a dual-luciferase reporter assay. The results of luciferase detection after transfection showed that the transcriptional activity of recombinant pGL3-*TFPI2* plasmid was significantly higher compared to the pGL3-Basic control vector (fold change = 5, *P* = 0.005, Figure 2B).

**Figure 4:**
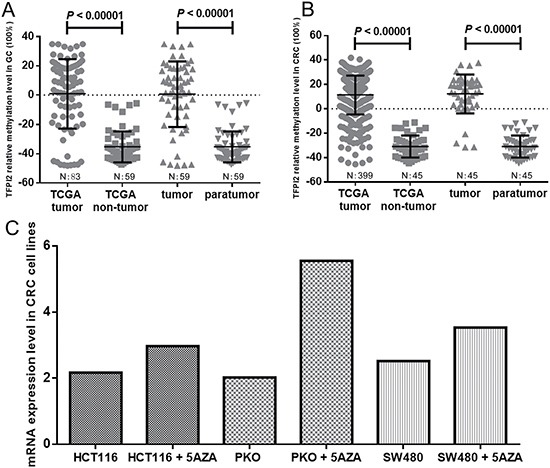
Analysis of TFPI2 gene methylation and mRNA expression in public databases Comparisons of TFPI2 methylation levels between tumor tissues and adjacent non-tumor tissues in (**A**) GC patients and (**B**) CRC patients from TCGA database. The data of TCGA tumor and TCGA non-tumor were extracted from 83 GC patients and 59 non-tumor patients in Figure 4A, 399 CRC patients and 45 non-tumor patients in Figure 4B, respectively. Tumor and paratumor were proceed from the same layer. N: number. Statistical values and the bars were presented as median with interquartile range. (**C**) The changes of mRNA expression levels in CRC cell lines (HCT116, PKO and SW480) with and without 5′-AZA-deoxycytidine treatment from GEO database (accession number GSE32323).

The Cancer Genome Atlas (TCGA) database also validated the percentage of methylated reference (PMR) of *TFPI2* gene in tumor tissues was higher than that in non-tumor adjacent tissues. Our data mining study showed that the relative methylation level was significantly higher in GC and CRC tissues (Figure 4A and 4B). Moreover, *TFPI2* methylation was inversely correlated with gene expression in both GC and CRC (cg12973591, GC: r = –0.447, CRC: r = –0.225; cg22799321, GC: r = –0.426, CRC: r = –0.190). Besides, using the data from TCGA, we calculated the correlation between the 10 CG sites in the CpG island (cg12973591, cg22799321, cg20230721, cg17338208, cg26739865, cg24531255, cg23686014, cg23141855, cg14377593, cg2241153) in CRC tissues and non-tumor tissues, respectively. High correlation was observed between methylation levels of these 10 CG sites (*P* < 0.00001, data not shown). We also calculated the correlation between the 4 CG sites in the CpG island (cg22799321, cg23686014, cg23141855, and cg14377593) in GC tissues and non-tumor tissues, respectively. High correlation was also observed among the methylation levels of these 4 CG sites (*P* < 0.00001, data not shown).

Using the data of Gene Expression Omnibus (GEO) database (GSE32275), we focused on 5 CG sites (cg22799321, cg23686014, cg23141855, cg14377593, and cg19784477) in the CpG island. Our results showed that DNA methylation level dropped after 5′-AZA-deoxycytidine treatment (Supplementary Figure 1). Using the data of GEO database (GSE32323), we found that *TFPI2* expression in three CRC cell lines (HCT116, RKO and SW480) was increased after 5′-AZA-deoxycytidine treatment (fold change > 1.37, Figure 4C). Therefore, *TFPI2* was likely to be hypermethylated in the cell lines, which exerted a potential suppressor on gene expression.

## DISCUSSION

In the current study, we compared the methylation level of *TFPI2* among the GC and CRC and the adjacent non-tumor tissues, and colorectal normal tissues. *TFPI2* hypermethylation was found in 85 out of 114 (75%) GC tissues and 61 out of 80 (76%) CRC tissues. Similarly, 52 out of 59 (88%) GC tissues and 45 out of 45 (100%) CRC tissues existed *TFPI2* hypermethylation in TCGA database. *TFPI2* methylation levels in CRC tissues were also significantly higher than those in colorectal normal tissues. Further data mining study of GEO data indicated that *TFPI2* was likely to be hypermethylated in the CRC cell lines. The above findings suggested an important role of *TFPI2* hypermethylation in the detection of GC and CRC.

In addition, although *TFPI2* hypermethylation has been reported in many human cancers, there existed some highlights in our study. Firstly, most of studies applied MSP method, which is a qualitative approach with a low sensitivity in methylation detection [26]. We have chosen a quantitative method which has been considered as a more suitable application in molecular diagnosis [16, 27]. Secondly, previous studies were involved with a relative fewer GC samples (ranging from 18 to 73) [19, 20]. In contrast, our GC sample size is 114. Thirdly, we further evaluated the regulation mechanism of *TFPI2* methylation on gene expression. A dual-luciferase reporter gene assay showed an enhanced transcriptional activity of *TFPI2* cloned fragment. Moreover, GEO data indicated that *TFPI2* expression was significantly increased in three CRC cell lines (HCT116, PKO and SW480) after 5′-AZA-deoxycytidine treatment. Our results helped us gain an insight into the role of *TFPI2* methylation in GC and CRC diagnosis.

Gastrointestinal malignant cancer was a genetically and phenotypically heterogeneous disease. Several distinct types of biomarkers have been studied in the diagnosis of cancer. Unfortunately, early detection of gastrointestinal malignant cancer has still been hindered by the absence of effective biomarkers. For example, carcinoembryonic antigen (CEA), as one of the most widely used tumor markers worldwide, played a role in cancer invasion and metastasis [28]. Fletcher has pointed out that CEA sensitivity and specificity were not high, particularly for early stages of disease, such as a sensitivity of 36% and a specificity of 87% in screening for colorectal cancer [29]. And Hibi *et al.* showed that the detection rate of colorectal cancer by using *TFPI2* methylation, CEA and CA19-9 was 18%, 33% and 17%, respectively [30]. Combined use of AFP, CEA, CA125 and CAl9-9 for the diagnosis of GC had a relatively low AUC of 0.667 (sensitivity: 0.403; specificity: 0.932) [31]. Thus, conventional plasma proteins may lack enough sensitivity in clinical detection of GC and CRC. Epigenetic biomarkers may serve as a tool to diagnose cancer and predict therapeutic effect and prognosis [32]. The methylation status of a primary tumor could be discovered by qMSP in advance, which could be used as a cancer screening method in principle [16]. Recently, many epigenetic biomarkers have been studied in cancer diagnosis, including *hMLH1*, *E-cadherin*, *CDKN2A*, *CDKN2B* and *APC* [33] in GC and *SEPT9*, *SFRP2*, *THBD*, *SDC2*, *VIM* and *FBN1* in CRC [34]. Nevertheless, few biomarkers were actually used as auxiliary diagnosis of tumor in the clinic due to its low sensitivity. For example, Hibi *et al.* found only 10% GC samples and 18% CRC samples exhibited *TFPI2* methylation [16, 27]. Our qMSP-based study showed that *TFPI2* hypermethylation yielded a high AUC of 0.762 (sensitivity: 68%; specificity: 83%) in GC and 0.759 (sensitivity: 61%; specificity: 84%) in CRC. In addition, the biomarker should be required to distinguish the early/late stages to accurately detect GC and CRC. According to our results, no statistically significant correlation was found between *TFPI2* methylation and the stages of GC and CRC patients. Hence, combining methylation biomarkers with other biomarkers might further improve the diagnostic accuracy [14].

Studies have reported that GC and CRC share some molecular characteristics including microsatellite instability, hypermethylation of tumor suppressor genes, and genetic mutations [35, 36]. And in addition, GC has very similar histopathology to CRC [37]. In the current study, we compared the *TFPI2* methylation levels between GC and CRC tumor samples, and no significant difference was found (*P* = 0.569). Our results showed that *TFPI2* hypermethylation has a similar diagnostic value for GC and CRC.

In the previous study, *TFPI2* methylation could reduce expression in canine Diffuse Large B-cell lymphomas [38] and esophageal squamous cell carcinoma [39]. *TFPI2* transcription restored in GC cells after treatment with 5′-AZA-deoxycytidine [20]. Our data mining of the public databases discovered there was an inverse correlation between *TFPI2* methylation and gene expression in different cancers. Meanwhile, *TFPI2* transcription in three CRC cell lines increased after 5′-AZA-deoxycytidine treatment in GEO database. In addition, our dual luciferase reporter assay showed a significantly higher activity in the recombinant strain with pGL3-*TFPI2* plasmid, suggesting that the *TFPI2* fragment was able to regulate gene expression.

Two familiar mechanisms of DNA methylation on transcriptional repression has been reported [40]. DNA methylation interfered with the combination of transcription factors and cis-element, which inhibited gene expression. In addition, methyl-CpG-binding protein altered the chromatin structure through recruiting the co-repressor complex. According to UCSC Genome Browser, the selected fragment in the luciferase study overlapped with multiple transcription factor binding sites, including CCCTC binding factor (CTCF) which is known to play an unusual role in different aspects of gene regulation including promoter activation and repression, hormone-responsive gene silencing, methylation-dependent chromatin insulation and genomic imprinting [41]. We hypothesized that *TFPI2* was likely to be hypermethylated in GC and CRC, which exerted a potential suppressor on gene expression through inhibiting transcription factor binding. However, the exact regulatory mechanism should be explored in the future.

*TFPI2* promoter was reported to be activated by *P14ARF* in a p53-independent manner that relied upon c-JUN, SP1, and JNK activity, which could reduce aggressive phenotypes associated with necrotic tumors [42]. And the expression of *TFPI2* had a decreasing trend with tumor progression of cervical cancer through inhibiting tumor cell apoptosis and angiogenesis [43]. Therefore, vascular thrombosis, hypoxia and necrosis induced by *TFPI2* low-expression could be the main reasons for aggressive phenotypes in gastric and colorectal cancer pathways. Further study should be explored in gastric and colorectal cancer pathways.

What's more, *TFPI2* lower-expression markedly inhibited its growth and metastasis *in vivo* by regulating pericellular extracellular matrix remodeling and angiogenesis [44]. Although *TFPI2* was shown to reduce the cell proliferation rate in various human tumors, Lai *et al.* found that *TFPI2* expression restoration after 5′-AZA-deoxycytidine treatment almost had no effect on suppressed cell proliferation and promoted cell apoptosis *in vitro* [45]. Therefore, the role of *TFPI2* in cell proliferation and apoptosis may be complicated. Functional studies *in vitro* and in nude mice model should be performed in the future.

There are the following aspects of main limitations in our study to be noted. We only obtained tumor tissues in the current study. Since DNA methylation as a biomarker was more effective in serum than that in tissues [46], the diagnostic value of *TFPI2* hypermethylation in serum should be assessed in the future. Due to the limitation of CRC cell lines, we only performed the transfection of *TFPI2* CpG island region pGL3-Promoter vectors in HEK293T cell line, and future study in CRC cell lines should be performed to better understand the activity of the chosen promoter region. Due to the limitation in primer design, only 2 CpG sites were evaluated to represent *TFPI2* methylation. However, a more comprehensive research covering more regions among larger number of samples should be carried out in the future. In addition, although we utilized GEO data to further explore the potential regulatory mechanism between DNA methylation and gene expression, more studies should be carried out.

In summary, our studies suggested that *TFPI2* hypermethylation might be an useful diagnostic biomarker for GC and CRC. Further studies on the detailed mechanisms of *TFPI2* hypermethylation in the carcinogenesis should be explored.

## MATERIALS AND METHODS

### Tissue samples

Frozen tumor tissues and their paired adjacent normal tissues (5 cm away from the tumor lesion) were obtained from 114 GC patients and 80 CRC patients during surgery. We also collected 22 colorectal normal tissues after a diagnostic endoscopy. Pathological examination was performed for the diagnosis of all the patients. Hematoxylin and eosin (H&E) stained slides were used to determine representative areas of invasive tumor. There are 80% of cancer cells presenting in each sample, which were examined by microscope. No tumor cells exist in the adjacent sample, and adjacent tissue was chosen from the same block as the tumor [47]. Besides, tumor and non-tumor samples proceed from the same layer. All the clinical information (including age, gender, disease stage, tumor differentiation and metastasis) was extracted from the medical records. Permission for the study was given by Human Research Ethics Committee at Shaoxing People's Hospital and Zhejiang Cancer Hospital. Written informed consent was obtained from each participant.

### DNA extraction and bisulphite conversion

Isolated DNA was extracted from frozen tissues by E.Z.N.A.^™^ Tissue DNA Kit (Omega Bio-Tek, Norcross, GA). Nanodrop2000 spectrophotometer (Thermal Scientific Co. Ltd., Wilmington, USA) was operated to measure DNA concentrations. And 500 ng DNA was used for bisulphite conversion. EZ DNA Methylation-Gold Kit ^™^ (Zymo Research, Orange, USA) was used to convert unmethylated cytosines into uracils correspondingly, while the methylated cytosines remained in the reaction.

### Quantitative methylation specific polymerase chain reaction (qMSP) method

*ACTB* was chosen as the internal reference to correct the differences in both quality and quantity between samples. Human sperm DNA was methylated as the positive control by excess SssI methyltransferase (Thermo Fisher Scientiific, Uppsala, Sweden). All the bisulphite-modified DNA was used as the template in qMSP assays, which were carried out in 384-well plates in the LightCycler 480 (Roche, Basel, Switzerland). The final reaction system contained 10 μL SYBR Green I Master (Roche, Basel, Switzerland), 0.5 μL forward primer (10 μm), 0.5 μL reverse primer (10 μm), 1.0 μL templates and 8 μL ddH_2_O. PCR was conducted under the following conditions: 1 cycle at 95°C for 10 min followed by 45 cycles at 95°C for 20 sec, 58°C for 20 sec, 72°C for 30 sec; 1 cycle for the melting curve analysis at 95°C of 15 sec, 60°C of 1 min; a final cooling stage at 40°C for 10 min. The qMSP primer sequences were as follows: *TFPI2* (88 bp), 5′-GAAATTGTTGGCGTTGTTT-3′ (forward) and 5′-CCTACTTCTCCGTTACTACT-3′ (reverse); *ACTB* (133 bp), 5′-TGGTGATGGAGGAGGTTTAGTAAGT-3′ (forward)and 5′-AACCAATAAAACCTACTCCTCCCTT AA-3′ (reverse). The PMR value of *TFPI2* was used to represent gene expression. And the PMR in each sample was calculated by 2^−ΔΔCt^ quantification approach, in which ΔΔCt = sample DNA (Ct _target gene_ – Ct _ACTB control_) − fully methylated DNA (Ct _target gene_ – Ct _ACTB control_) [48]. qMSP products were subjected to the Qsep100 DNA fragment analyzer (Bioptic, Taiwan, China), and they could be visualized by multiple objective straps in Gel-view format as previously described [49].

### Dual-luciferase reporter assay

Dual-luciferase reporter system is a standard method that utilized firefly and renilla luciferase to study the promoter activity of target fragment [50]. Firefly and renilla luciferases are two commonly used reporters and are introduced into cells by co-transfecting cells with a luciferase reporter construct and an internal control vector to quantifiable luminescence [51]. The purpose of the dual luciferase assay in the current study was to show that the chosen regions might be functional in the regulation of gene expression. HEK293T cell line has a high success rate of transfection, and it is commonly used for plasmid transfection [52]. Therefore, we used the HEK293T cell line instead of CRC cell lines to perform the dual-luciferase reporter assay. Human embryonic kidney HEK293T cell line was cultured as described previously [53]. A fragment of *TFPI2* CpG island (+538 bp to +938 bp) was chemically synthesized and was digested with XhoI and KpnI (New England Biolabs, Ipswich, MA). The target DNA fragment, purified by Cycle Pure Kit (Omega, Norcross, GA, USA), was ligated to pGL3-Basic vector using DNA Ligation Kit (TaKaRa, Japan). The empty pGL3-Basic vector (Promega, Madison city, WI, USA) was used as the negative control, and the pGL3-Basic vector containing an SV40 promoter upstream of the luciferase gene, was used as the positive control.

Human HEK293T cells in the exponential growth phase were rinsed twice with phosphate buffer saline (PBS), treated with trypsin, and supplemented with dulbecco's minimum essential medium (DMEM) with 10% fatal bovine serum (FBS). The cells were calculated on 24-well plates. After 12 hours, 0.5 × 10^5^ cells per well were transfected with recombinant plasmid according to the manufacturer's protocol (TransLipid HL Transfection Reagent, TransGen Biotech, Beijing, China). After 4–8 hours, medium was changed by fresh DMEM with 10% FBS. After 36 hours of 293T cells transfection, renilla and firefly luciferase activity was measured by SpectraMax 190 (Molecular Devices, Sunnyvale, USA). Reporter gene activity was assessed according to the manufacturer's protocol (Dual-Luciferase^®^ Reporter Assay Systems, Promega, Madison city, WI, USA).

### Data mining study

We extracted the 27K array data of 83 GC patients and 450K array data of 399 CRC patients from the TCGA database. First, we compared the *TFPI2* methylation levels between the 83 GC tissues and 59 non-tumor tissues. And *TFPI2* methylation levels also were compared between the 399 CRC tissues and 45 non-tumor tissues. Next, 59 paired GC samples and 45 paired CRC samples were chosen to validate the results.

DNA methylation is a reversible biochemical process [54], and 5′-AZA-deoxycytidine could serve as an inhibitor of DNA methylation [55]. 5′-AZA-deoxycytidine treatment is often used to restore the expression levels of genes which were expressed in low levels due to their hypermethylated promoters [56]. We retrieved the expression data derived from the Gene Expression Omnibus (GEO) database (www.ncbi.nlm.nih.gov/geo, accession no. GSE32323). We compared on the expression changes of *TFPI2* in three CRC cell lines (HCT116, RKO and SW480) with and without 5′-AZA-deoxycytidine treatment.

### Statistical analysis

All the data were analyzed by SPSS 18.0 software (SPSS Inc, Chicago, IL, USA) and GraphPad Prism 5.0 Software (GraphPad Software, La Jolla, CA). Data were presented as median (interquartile range). A nonparametric Mann-Whitney *U* test was used to assess the methylation differences between tumor tissues and normal tissues. Spearman's correlation was used to evaluate the correlation between *TFPI2* methylation level and clinical characteristics. Receiver operating characteristic (ROC) analysis showed the diagnostic value of *TFPI2* methylation. A two-side *P* < 0.05 was defined to be statistically significant.

## SUPPLEMENTARY MATERIALS AND FIGURES



## Abbreviations

*TFPI2*: tissue factor pathway inhibitor 2
GC: gastric cancer
CRC: colorectal cancer
qMSP: quantitative methylation-specific polymerase chain reaction
PMR: percent of methylated reference
CpG: cytosine-phosphate-guanine
PPV: positive predictive value
NPV: negative predictive value
GEO: Gene Expression Omnibus
PBS: phosphate buffer saline
DMEM: dulbecco's minimum essential medium
FBS: fatal bovine serum
HM27K: Illumina Human Methylation 27K
HM450K: Illumina Human Methylation 450K
TCGA: The Cancer Genome Atlas
RMA: robust multi-array average
ROC: Receiver operating characteristic
