# Supplementary material for: The role of TFPI2 hypermethylation in the detection of gastric and colorectal cancer
Source: Oncotarget. 2017 Sep 20;8(48):84054–65. doi: 10.18632/oncotarget.21097 (PMC5663576; doi:10.18632/oncotarget.21097)
Supplement: Supplementary file 1 [file oncotarget-08-84054-s001.pdf]

The role of *TFPI2* hypermethylation in the detection of gastric and colorectal cancer

SUPPLEMENTARY MATERIALS

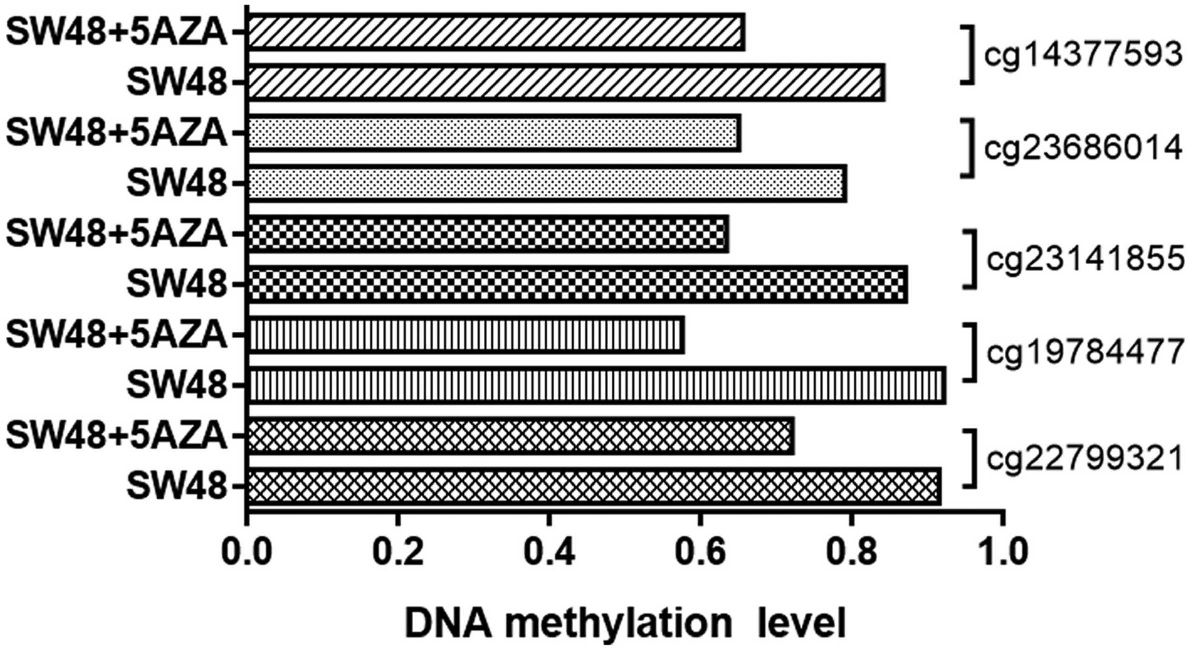

Supplementary Figure 1: The changes of DNA methylation levels in CRC cell line (SW48) with and without 5′-AZA-deoxycytidine treatment from GEO database (accession number GSE32275).
